# Cryo‐EM structure and biochemical analyses of the nucleosome containing the cancer‐associated histone H3 mutation E97K

**DOI:** 10.1111/gtc.13143

**Published:** 2024-07-07

**Authors:** Tomoaki Kimura, Seiya Hirai, Tomoya Kujirai, Risa Fujita, Mitsuo Ogasawara, Haruhiko Ehara, Shun‐ichi Sekine, Yoshimasa Takizawa, Hitoshi Kurumizaka

**Affiliations:** ^1^ Laboratory of Chromatin Structure and Function, Institute for Quantitative Biosciences, The University of Tokyo Tokyo Japan; ^2^ Department of Biological Sciences Graduate School of Science, The University of Tokyo Tokyo Japan; ^3^ RIKEN Center for Biosystems Dynamics Research Yokohama Japan

**Keywords:** cancer, chromatin, cryo‐EM, epigenetics, histone mutation, nucleosome

## Abstract

The Lys mutation of the canonical histone H3.1 Glu97 residue (H3E97K) is found in cancer cells. Previous biochemical analyses revealed that the nucleosome containing the H3E97K mutation is extremely unstable as compared to the wild‐type nucleosome. However, the mechanism by which the H3E97K mutation causes nucleosome instability has not been clarified yet. In the present study, the cryo‐electron microscopy structure of the nucleosome containing the H3E97K mutation revealed that the entry/exit DNA regions of the H3E97K nucleosome are disordered, probably by detachment of the nucleosomal DNA from the H3 N‐terminal regions. This may change the intra‐molecular amino acid interactions with the replaced H3 Lys97 residue, inducing structural distortion around the mutated position in the nucleosome. Consistent with the nucleosomal DNA end flexibility and the nucleosome instability, the H3E97K mutation exhibited reduced binding of linker histone H1 to the nucleosome, defective activation of PRC2 (the essential methyltransferase for facultative heterochromatin formation) with a poly‐nucleosome, and enhanced nucleosome transcription by RNA polymerase II.

## Graphical abstract

Cryo‐EM structure of the nucleosome containing the H3 E97K mutation was determined. The H3 E97K mutation enhances the flexibility of the nucleosomal DNA ends. The H3 E97K mutation affects the linker histone H1 binding, RNAPII transcription, and methyltransferase activity of PRC2.

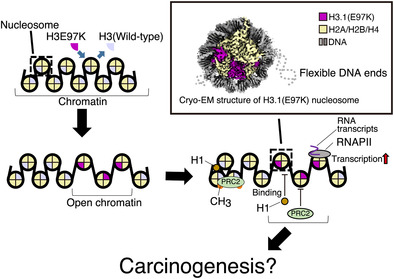

## 1 INRODUCTION

In eukaryotes, genomic DNA is tightly compacted by the nucleosome, in which roughly 150 base pairs (bp) of DNA are wrapped around a histone octamer composed of two each of histones H2A, H2B, H3, and H4 (Luger et al., 1997). The nucleosomes form a beads‐on‐a‐string configuration with linker DNAs (Kornberg, 1974; Olins & Olins, 1974). These poly‐nucleosome fibers are further compacted into a higher order architecture termed chromatin (Wolffe, 2000). In chromatin, genome activities, such as transcription, replication, recombination, and repair, are generally suppressed and epigenetically regulated through chromatin structure.

Single amino acid substitutions of histones substantially affect the structure and stability of the nucleosome (Flaus et al., 2004; Kruger et al., 1995; Kurumizaka & Wolffe, 1997; Muthurajan et al., 2004), suggesting that these alterations may disturb proper gene regulation in chromatin (Kruger & Herskowitz, 1991; Peterson et al., 1991; Sternberg et al., 1987). In mammalian cells, such improper gene regulation due to histone mutations may cause carcinogenesis. Consistent with this idea, mutations in histone genes have been identified in many cancer cells (Nacev et al., 2019). Among them, cancer‐related mutations of H2B Glu76 to Lys and H2A.Z Arg80 to Cys reportedly induce substantial alterations in the nucleosome structure and stability (Arimura et al., 2018). In addition, the canonical H3.1 Glu97 to Lys (H3E97K) mutation has been found in cancer cells, and reportedly reduces the nucleosome stability (Arimura et al., 2018; Bagert et al., 2021). However, the mechanism by which this H3 mutation destabilizes the nucleosome has remained elusive, because the structure of the nucleosome containing H3E97K has not been determined due to its extreme instability.

In the present study, we successfully reconstituted the nucleosome containing H3E97K and determined its cryo‐electron microscopy (cryo‐EM) structure. We also tested the biochemical consequences of the H3E97K mutation for linker histone H1 binding, the methyltransferase activity of Polycomb Repressive Complex 2 (PRC2), and the nucleosome transcription by RNA polymerase II (RNAPII).

## 2 RESULTS AND DISCUSSION

### 2.1 Cryo‐EM structure of the nucleosome containing the H3E97K mutation

**FIGURE 1:**
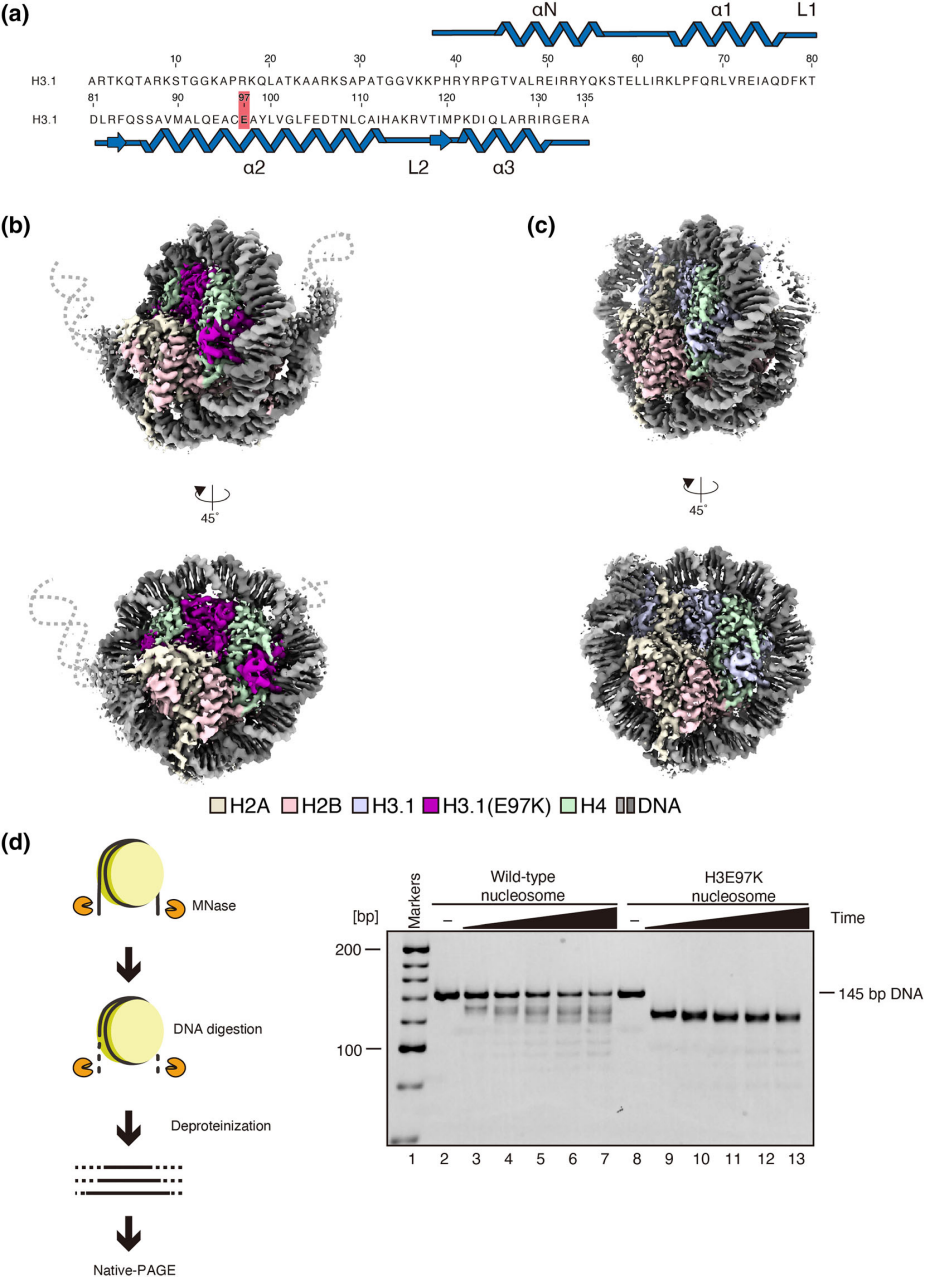
Cryo‐EM structure of the H3E97K nucleosome. (a) Amino acid sequence of human H3.1. The H3 Glu97 residue is highlighted in red. (b) Cryo‐EM map of the H3E97K nucleosome. Histones H3.1 E97K, H2A, H2B, and H4 are colored purple, yellow, pink, and green, respectively. DNA is colored gray. The dashed lines indicate possible disordered DNA regions. (c) Cryo‐EM map of the wild‐type nucleosome. Histones H3.1, H2A, H2B, and H4 are colored blue, yellow, pink, and green, respectively. DNA is colored gray. (d) Schematic diagram of micrococcal nuclease assay (left panel). Representative gel image of the micrococcal nuclease assay (right panel). The nucleosomes containing wild‐type H3 (lanes 2–7) or the H3E97K mutant (lanes 8–13) were treated with MNase for 0 min (lanes 2 and 8), 3 min (lanes 3 and 9), 6 min (lanes 4 and 10), 9 min (lanes 5 and 11), 12 min (lanes 6 and 12), and 15 min (lanes 7 and 13). The resulting DNA fragments were fractionated by native‐PAGE, followed by EtBr staining. The reproducibility of the results was confirmed by two additional, independent experiments (shown in Figure S4).

The H3E97K mutation in canonical H3.1 has been identified in cancer cells (Figure 1a). A previous biochemical study revealed that the nucleosome containing the H3E97K mutation (the H3E97K nucleosome) is extremely unstable with the α‐satellite DNA sequence and may be difficult to crystallize (Arimura et al., 2018). To overcome this problem, we reconstituted the H3E97K nucleosome with the Widom 601 DNA, which is known to form stable nucleosomes (Lowary & Widom, 1998). As shown in Figure S1, the H3E97K nucleosome was successfully reconstituted with the Widom 601 sequence (Figure S1a–c). Consistent with the previous results, the H3E97K nucleosome with the Widom 601 DNA also exhibited substantial thermal instability as compared with the wild‐type nucleosome (Figure S1d,e).

We then determined the cryo‐EM structure of the H3E97K nucleosome with the Widom 601 DNA (Figure 1b and Figure S2). For comparison, we also determined the cryo‐EM structure of the wild‐type nucleosome with the Widom 601 DNA (Figure 1c and Figure S3). We found that the entry/exit DNA regions of the H3E97K nucleosome are flexibly disordered, as compared with those of the wild‐type nucleosome (Figure 1b,c). This finding was consistent with the susceptibility assay for micrococcal nuclease (MNase), which preferentially digests the DNA detached from the histone surface in the nucleosome (Noll et al., 1975). As shown in Figure 1d, the DNA ends of the H3E97K nucleosome were rapidly degraded by MNase, as compared with the wild‐type nucleosome, indicating the enhanced accessibility. In the H3E97K nucleosome, roughly 120 base pairs of DNA were protected from MNase digestion (Figure 1d). This is consistent with the disordered DNA ends of the H3E97K nucleosome structure.

**FIGURE 2:**
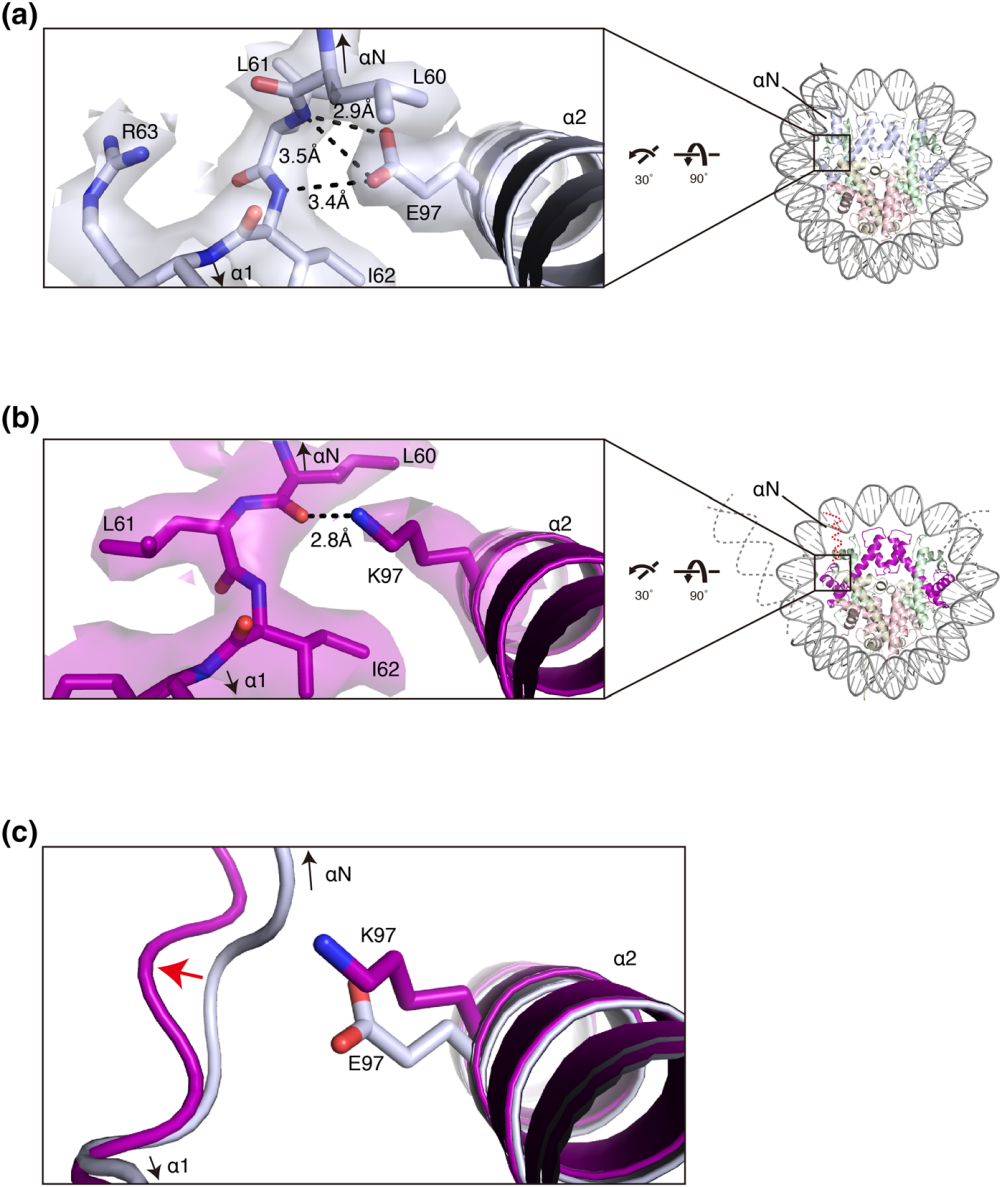
Structural comparison of the H3E97K nucleosome with the wild‐type nucleosome around H3 residue 97. (a) Close‐up view around the residue at position 97 in the wild‐type nucleosome (left panel). The enlarged region of the wild‐type nucleosome is indicated with rotation angles (right panel). The cryo‐EM map corresponding to the Glu97 residue is overlaid on the atomic model. The dashed lines indicate possible hydrogen bonds. (b) Close‐up view around the residue at position 97 in the H3E97K nucleosome (left panel). The enlarged region of the H3E97K nucleosome is indicated with rotation angles (right panel). The cryo‐EM map corresponding to the Lys97 residue is overlaid on the atomic model. The black dashed line indicates a possible hydrogen bond. The gray dashed lines and the red dashed line indicate putatively disordered DNA regions and a possibly disordered H3 αN helix, respectively. (c) Structural comparison around position 97 of H3 in the wild‐type and H3E97K nucleosomes. The wild‐type H3 and H3E97K mutant are colored light blue and purple, respectively. The red arrow indicates the local distortion of the H3 structure in the H3E97K nucleosome.

In the wild‐type nucleosome, the sidechain carbonyl group of the H3 Glu97 residue forms hydrogen bonds with the main‐chain N atoms of the H3 Leu61 and Ile62 residues (Figure 2a). In contrast, in the H3E97K nucleosome structure, the sidechain ε^NH3^ group of the H3 Lys97 residue apparently forms a hydrogen bond with the main‐chain carbonyl group of the H3 Leu60 residue (Figure 2b). Accordingly, the backbone geometry of H3 around these residues is distorted in the H3E97K nucleosome (Figure 2c). This structural difference changes the location of the H3 αN helix (amino acid residues 44–57), which plays an important role to capture the entry/exit DNA regions at the nucleosomal DNA ends, and may induce the DNA end flexibility in the H3E97K nucleosome (Figure 2a–c).

### 2.2 The H3E97K mutation reduces the linker histone H1 binding to the nucleosome

**FIGURE 3:**
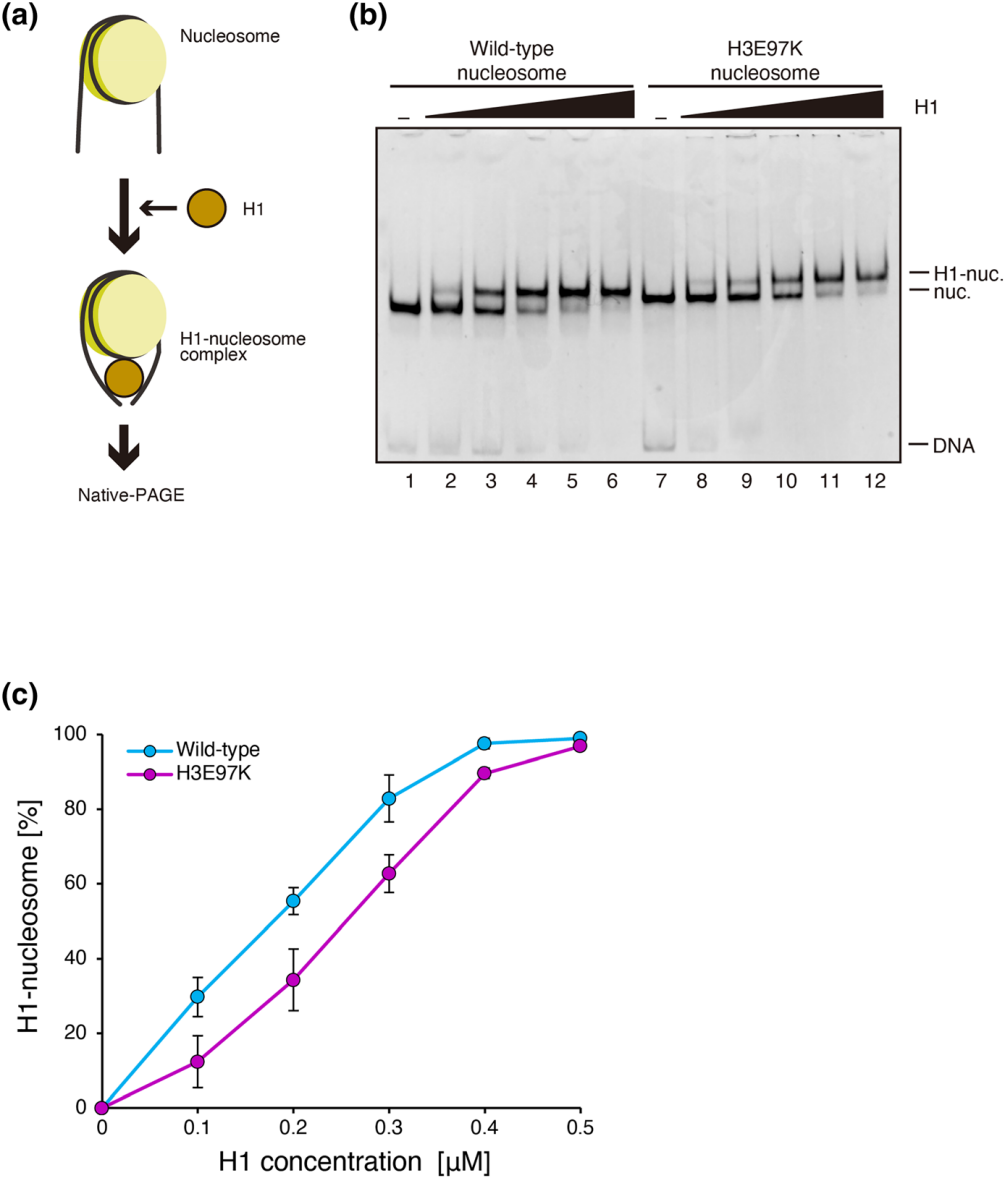
Linker histone H1 binding in the H3E97K nucleosome. (a) Schematic diagram of the H1 binding assay. (b) A representative gel image of the H1 binding assay. The nucleosome (0.1 μM) containing wild‐type H3 (lanes 1–6) or the H3E97K mutant (lanes 7–12) was mixed with the indicated amounts of the linker histone H1.2 (0 μM: Lanes 1 and 7; 0.1 μM: Lanes 2 and 8; 0.2 μM: Lanes 3 and 9; 0.3 μM: Lanes 4 and 10; 0.4 μM: Lanes 5 and 11; 0.5 μM: Lanes 6 and 12). After an incubation at 37°C, the H1‐nucleosome complexes were analyzed by native‐PAGE with EtBr staining. The reproducibility of the results was confirmed by two additional, independent experiments (shown in Figure S4c,d). (c) Graphical representation of the H1 binding assay. The band intensities corresponding to the unbound nucleosome were quantified, and the rate of H1‐nucleosome complex formation was plotted against the concentration of H1. The error bars indicate standard deviations (*n* = 3). Purple and light blue circles indicate experiments with the H3E97K nucleosome and the wild‐type nucleosome, respectively.

Linker histones, such as H1, bind the linker and dyad DNA regions of the nucleosome and tie the linker DNA (Bednar et al., 2017; Zhou et al., 2021). Therefore, H1 is considered to play an important role in facilitating a higher chromatin conformation by restricting the linker DNA orientation and flexibility. To test the H1 binding to the H3E97K nucleosome, we performed the gel mobility shift assay. To do so, we reconstituted the nucleosomes with a 193 base‐pair DNA, which produces 23 base‐pair linker DNAs at both ends of the nucleosome (Figure 3a). We found that the H3E97K nucleosome substantially reduced the H1 binding (Figure 3a–c, and Figure S5). This may be due to the enhanced entry/exit DNA flexibility observed in the H3E97K nucleosome (Figure 1b). Therefore, the chromatin region with the H3E97K mutant may tend to incorporate less H1.

### 2.3 The poly‐nucleosome containing the H3E97K mutation is defective in stimulating the methyltransferase activity of PRC2

**FIGURE 4:**
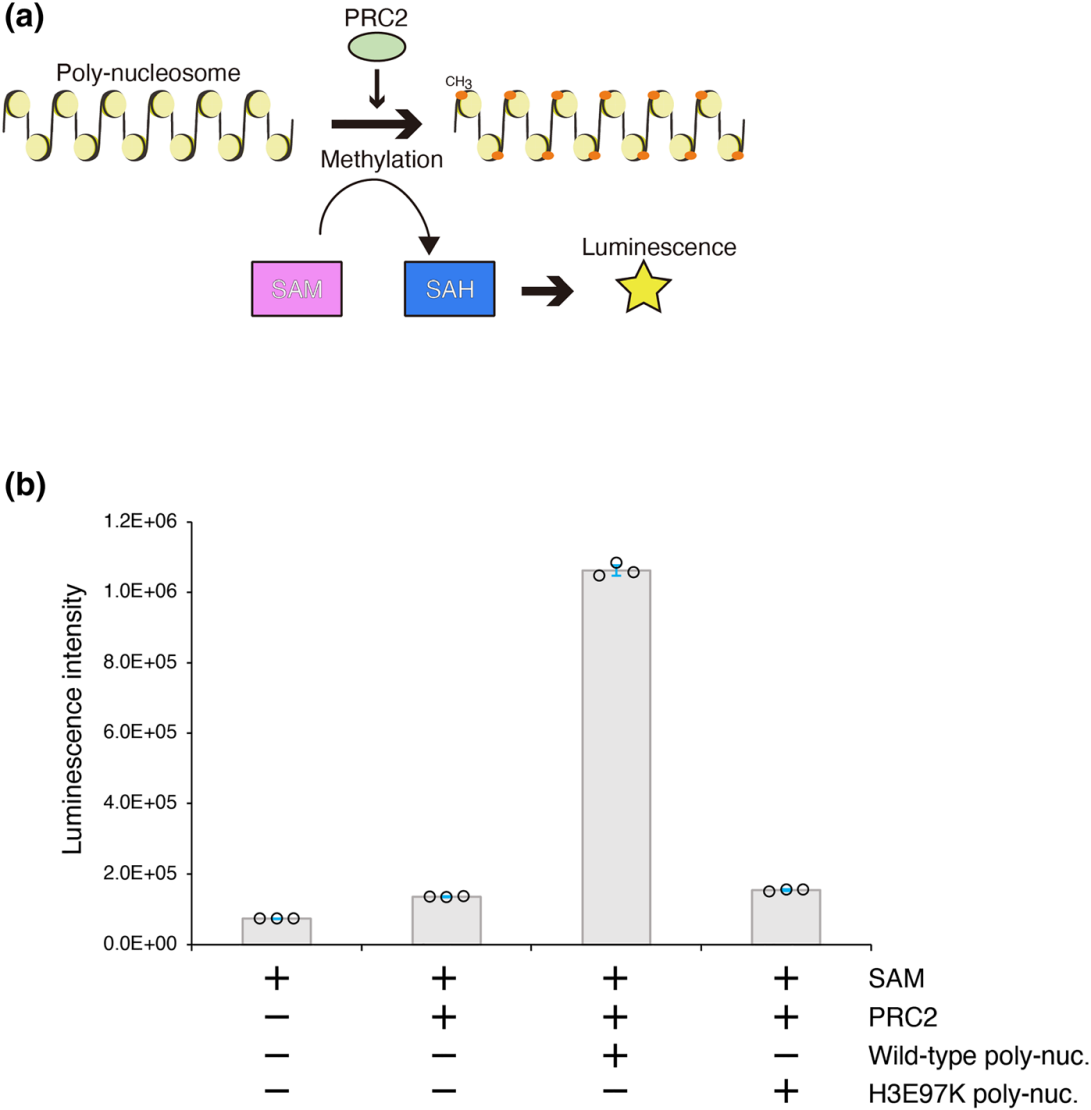
Defective stimulation of the PRC2 methyltransferase activity by the poly‐nucleosome containing the H3E97K mutant. (a) Schematic diagram of the PRC2 methyltransferase assay. The wild‐type or H3E97K poly‐nucleosome was incubated at 30°C in the presence of the PRC2 core enzyme complex and S‐adenosylmethionine (SAM). The PRC2 complex promotes the H3 methylation of a poly‐nucleosome, using SAM as the methyl‐group donor. After the reaction, the resulting S‐adenosylhomocysteine (SAH) was measured by detecting luminescence, using an MTase‐Glo™ Methyltransferase Assay Kit (Promega), and the methyltransferase activity of PRC2 was evaluated. (b) Graphical representation of the PRC2 methyltransferase assay. The bar graphs represent the luminescence intensity of the samples. The error bars indicate standard deviations (*n* = 3).

In facultative heterochromatin, the H3 Lys27 residue is methylated by PRC2, which plays crucial role in the H3 Lys27 methylation (Boyer et al., 2006; Bracken et al., 2006; Lee et al., 2006). The methyltransferase activity of PRC2 is reportedly stimulated by a poly‐nucleosome substrate with a compacted conformation, but not an extended conformation (Yuan et al., 2012). Therefore, we tested whether the H3E97K mutation affects the methyltransferase activity of PRC2 (Figure 4a). Consistent with the previous results, the methyltransferase activity of PRC2 was drastically stimulated by the wild‐type poly‐nucleosome (Figure 4b). In contrast, the poly‐nucleosome containing the H3E97K nucleosomes was quite defective in stimulating the PRC2 methyltransferase activity (Figure 4b). These results suggested that PRC2 may not promote the H3 Lys27 methylation of the poly‐nucleosome template containing the H3E97K nucleosome. The H3E97K poly‐nucleosome could form an extended conformation due to the flexibility of the nucleosomal entry/exit DNA, and this extended conformation may not be suitable for activating the PRC2 methyltransferase.

### 2.4 The H3E97K mutation enhances nucleosome transcription

**FIGURE 5:**
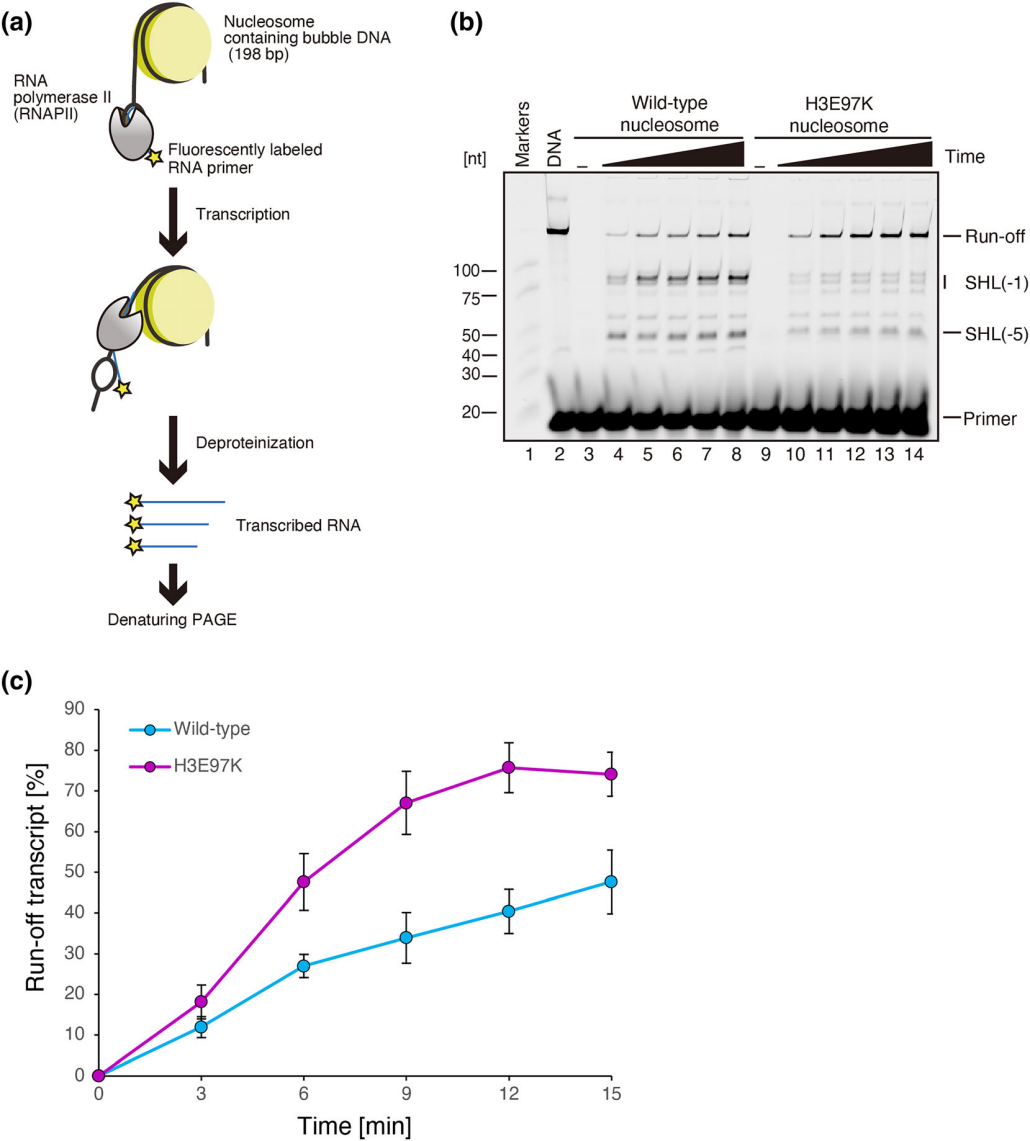
RNAPII transcription on the H3E97K nucleosome. (a) Schematic diagram of the RNA polymerase II transcription assay. (b) A representative gel image of the RNA polymerase II transcription assay. The nucleosome containing wild‐type H3 (lanes 3–8) or the H3E97K mutant (lanes 9–14) was incubated with RNAPII, TFIIS, and DY647 fluorescently labeled RNA primer at 30°C for 0 min (lanes 3 and 9), 3 min (lanes 4 and 10), 6 min (lanes 5 and 11), 9 min (lanes 6 and 12), 12 min (lanes 7 and 13), and 15 min (lanes 8 and 14). After the reaction, the samples were deproteinized, and the resulting elongated RNA transcripts were analyzed by urea denaturing‐PAGE with the detection of DY647 fluorescence. The reproducibility of the results was confirmed by two additional, independent experiments (shown in Figure S6c,d). (c) Graphical representation of the transcription assay. The band intensities of the run‐off transcripts were quantified. The run‐off transcripts (%) relative to that of the naked DNA template were plotted against the reaction time. The error bars indicate standard deviations (*n* = 3). Purple and blue circles indicate experiments with the H3E97K and wild‐type nucleosomes, respectively.

We finally tested the RNAPII transcription on the H3E97K nucleosome. To do so, we prepared the wild‐type and H3E97K nucleosomes containing a bubble region for the RNAPII start site on one linker DNA (Figure S6a,b). We then performed the nucleosome transcription assay in vitro (Figure 5a), in the presence of *Komagataella pastoris* RNAPII and transcription elongation factor IIS (TFIIS), as described previously (Ehara et al., 2019; Kujirai, Ehara, et al., 2018). Consistent with previous results (Ehara et al., 2019; Kujirai, Ehara, et al., 2018), the RNAPII paused at nucleosomal positions corresponding to superhelical locations (SHL) −5 and −1 (Figure 5b, lanes 4–8). SHL(−5) and SHL(−1) are defined as the nucleosomal DNA positions approximately 50 base pairs and 10 base pairs away from the dyad site, SHL(0), toward the RNAPII start site. In the H3E97K nucleosome, the RNAPII pausing at SHL(−5) and SHL(−1) positions was drastically reduced (Figure 5b, lanes 10–14). In addition, the run‐off RNA transcript, which is produced by RNAPII transcription to the end of the nucleosomal DNA substrate, was substantially increased in the H3E97K nucleosome as compared to that in the wild‐type nucleosome (Figure 5b,c). These may be a consequence of the unstable nature of the H3E97K nucleosome.

### 2.5 Conclusions and future perspectives

Our cryo‐EM analysis of the H3E97K nucleosome structure demonstrated that the entry/exit DNA regions at both nucleosomal DNA ends are drastically disordered by the defective DNA binding of H3. This may occur by intra‐molecular structural changes of H3 by the H3E97K mutation. Our biochemical analyses revealed that the H3E97K mutation reduced the linker histone H1 binding, consistent with the DNA flexibility at the entry/exit DNA regions of the H3E97K nucleosome. In addition, the poly‐nucleosome containing the H3E97K mutation was quite defective in stimulating the methyltransferase activity of PRC2, an essential methyltransferase for facultative heterochromatin formation. It has been reported that nucleosome proximity is an important factor for enhancement of the H3 Lys27 methylation by PRC2 (Yuan et al., 2012). Therefore, the poly‐nucleosome containing the H3E97K mutation may form more relaxed conformation as compared to the wild‐type poly‐nucleosome. This may cause the defective stimulation of the PRC2 activity with the H3E97K poly‐nucleosome. We also found that RNAPII transcription was enhanced in the H3E97K nucleosome, probably due to its unstable nature. The defective H1 binding, the reduced H3 Lys27 methylation, and the enhanced RNAPII transcription in the H3E97K nucleosome may result inappropriate gene regulation, and may be related to malignant transformation of cancer cells.

Our results reveal the structure of the nucleosome containing the H3E97K mutation, and explain the biochemical consequences of the H3E97K mutation on the nucleosome. These findings may provide important information for understanding the mechanisms of carcinogenesis by histone mutations.

## 3 EXPERIMENTAL PROCEDURES

### 3.1 Preparation of histone proteins and histone complexes

The human histones H2A, H2B, H4, H3.1, and H3.1 E97K mutant were purified as recombinant proteins, following the method outlined previously (Arimura et al., 2018; Kujirai, Arimura, et al., 2018). The H3–H4 and H2A–H2B complexes were reconstituted with lyophilized histones, which were purified as bacterially produced recombinant proteins. The resulting the H3–H4 and H2A–H2B complexes were further purified by Superdex 200 gel filtration chromatography (Cytiva).

### 3.2 Reconstitution and purification of nucleosomes

The nucleosomes with 145 base pairs of the Widom 601 DNA, 193 base pairs of the Widom 601 DNA, or 198 base pairs of the modified Widom 601 DNA (for RNAPII transcription assay) were reconstituted and purified, as described previously (Kujirai, Arimura, et al., 2018; Kujirai, Ehara, et al., 2018). Briefly, the nucleosomes containing the 145 base‐pair or the 193 base‐pair DNA were reconstituted by the salt‐dialysis method. For the nucleosome containing the 198 base‐pair DNA, the nucleosome was reconstituted with 153 base pairs of the modified Widom 601 DNA, and the 45 base‐pair DNA containing a mismatch bubble of 9 nucleotides was ligated with one end of the reconstituted nucleosome. These nucleosomes were purified with a Prep Cell apparatus (Bio‐Rad).

### 3.3 Thermal denaturation assay of nucleosomes

The thermal stabilities of the nucleosomes were evaluated following the method established previously (Taguchi et al., 2014). The purified nucleosomes with the 145 base‐pair DNA (22.3 pmol) were incubated in 19 μL volume of the reaction mixture (17 mM Tris‐HCl (pH 7.5), 4.25% glycerol, 100 mM NaCl, 0.85 mM dithiothreitol, and 5× SYPRO Orange [SIGMA‐Aldrich]). The temperature was raised from 25 to 95°C, at a rate of 1°C/min. The fluorescence of SYPRO Orange was measured with a StepOnePlus™ Real‐Time PCR system (Applied Biosystems). The fluorescence intensities were normalized as following formula: *F*(*T*)_normalized_ = [*F*(*T*) − *F*(26)]/[*F*(95) − *F*(26)], where F(T) represents the fluorescence signal intensity at a specific temperature.

### 3.4 Sample preparation for the cryo‐EM analysis

The nucleosome samples for cryo‐EM single‐particle analysis were prepared by the Gradient Fixation (GraFix) method (Stark, 2010). A 5 to 20% (w/v) sucrose gradient solution was prepared with low sucrose buffer (10 mM 4‐(2‐hydroxyethyl)‐1‐piperazine ethanesulfonic acid (HEPES)‐NaOH (pH 7.5), 1 mM dithiothreitol, 20 mM NaCl, and 5% sucrose) and high sucrose buffer (10 mM HEPES‐NaOH (pH 7.5), 1 mM dithiothreitol, 20 mM NaCl, 4% paraformaldehyde, and 20% sucrose) in Ultra‐Clear™ Centrifuge Tubes (344059; BECKMAN COULTER), using a Gradient Master 108 (SK Bio International). The purified nucleosome (0.4 nmol) was applied to the gradient solution and centrifuged for 16 h at 27,000 rpm and 4°C, using an SW41Ti rotor (Beckman Coulter). After the centrifugation, fractions (625 μL) were taken from the top of the gradient solution and fractionated by native polyacrylamide gel electrophoresis (PAGE) with ethidium bromide (EtBr) staining. Fractions containing nucleosomes were desalted with buffer, containing 20 mM HEPES‐KOH (pH 7.5), 0.2 μM zinc acetate, 50 mM potassium acetate, and 0.1 mM Tris(2‐carboxyethyl) phosphine (TCEP), using a PD‐10 column (Cytiva). The sample was then concentrated and applied onto Quantifoil Cu grids (R1.2/1.3. 200 mesh), which had been plasma cleaned using a H_2_/O_2_ gas mixture for 20 s in a Solarus II (Gatan). The grids were plunge frozen by vitrification using a Vitrobot Mark IV (Thermo Fisher Scientific) at 4°C and 100% humidity.

### 3.5 Cryo‐EM data collection

Images of the nucleosome samples were obtained by a Krios G4 cryo‐EM (Thermo Fisher Scientific) operated at 300 kV acceleration voltage, using a K3 direct detection camera and a BioQuantum GIF imaging filter (Gatan) with a slit width of 20 eV. Data acquisition of images was automated with the EPU software (Thermo Fisher Scientific). Micrographs were collected at a nominal magnification of 81,000× (a pixel size of 1.06 Å/pix) with an exposure time of 4.5 s and a dose of ~1.5 e^−^/Å^2^ per frame over 40 frames, and a defocus values range from −1.0 to −2.5 μm with an interval of 0.25 μm.

### 3.6 Image processing

In total, 7915 movies for the wild‐type nucleosome and 5806 movies for the H3E97K nucleosome were motion‐corrected with dose‐weighting, using MOTIONCOR2 (Zheng et al., 2017). The contrast transfer function (CTF) was estimated from the dose‐weighted micrographs with CTFFIND4 (Rohou & Grigorieff, 2015). The following data processing was conducted with Relion‐4.0 (Kimanius et al., 2021). The global resolution of the final map was estimated according to the Fourier shell correlation = 0.143 criterion (Scheres, 2016). For the wild‐type nucleosome, 235,657 particles were initially picked by LoG‐based auto‐picking from 400 micrographs. After excluding junk particles by 2D classification, 181,366 particles were selected for subsequent 3D classification. The cryo‐EM map of the *Xenopus laevis* nucleosome with the 145 base‐pair Widom 601 DNA (EMDB ID: EMD‐12900 [Wang et al., 2021]) was low‐pass filtered to 20 Å and employed as the initial reference model. After the 3D classification, 103,895 particles were selected to be used for Topaz training, and 3,591,830 particles were picked from the 7915 micrographs. After 2D classification and two rounds of 3D classification, a total of 2,366,250 particles were subjected to 3D auto‐refinement, followed by Bayesian polishing and CTF refinement. The refined map was sharpened with a *B*‐factor of −69.2 Å^2^. The resolution of the final map was estimated to be 2.38 Å.

For the H3E97K nucleosome, 74,752 particles were initially picked from 100 selected micrographs using LoG picker and subjected to 2D classification. Five good classes, containing 11,465 particles, were used for subsequent 2D reference‐based picking, and 6,153,691 particles were automatically picked from the 5551 micrographs. After removing junk particles by two rounds of 2D classification, 4,440,540 particles were subjected to the subsequent 3D classification. The ab initio model reconstructed by RELION‐4.0 was low‐pass filtered to 20 Å and used as the initial reference model. After the 3D classification, 1,899,818 particles were selected to be used for Topaz training, and 2,993,142 particles were picked from the 5806 micrographs. Subsequent 2D and 3D classification removed junk particles, resulting in the selection of 1,569,054 particles, which were subjected to 3D auto‐refinement, Bayesian polishing, and CTF refinement. The refined map was sharpened with a *B*‐factor of −69.3 Å^2^. The global resolution of the final map was estimated to be 2.69 Å.

### 3.7 Model building and refinement

The wild‐type nucleosome model was generated by combining the 145 base‐pair 601 DNA (PDB ID: 7OHC) and histones from the currently best‐resolved cryo‐EM nucleosome structure (PDB ID: 7VZ4). The histones and the DNA were placed into the density map by a UCSF Chimera (Pettersen et al., 2004) and phenix.dock_in_map (Liebschner et al., 2019). Among the two dyad‐related orientations of the nucleosomal DNA, the one with a higher model‐map correlation coefficient (CC) value was selected. The resulting model was real‐space refined against the EM map using Phenix (Liebschner et al., 2019), followed by manual editing using COOT (Emsley et al., 2010) and ISOLDE (Croll, 2018) to improve the geometric quality of the model and model‐to‐map fitting.

For building the H3E97K nucleosome model, the atomic coordinates of the wild‐type nucleosome modeled above were rigid‐body fitted into the density map of the H3E97K nucleosome by UCSF Chimera (Pettersen et al., 2004) and phenix.dock_in_map (Liebschner et al., 2019). The 97th Glu residue of H3 was adjusted to be the Lys residue by using the simple mutate tool in COOT (Emsley et al., 2010). To improve the fit of the nucleosomal DNA ends, interactive molecular dynamics flexible fitting was performed using ISOLDE (Croll, 2018). The resulting model was refined by phenix.real_space_refine (Liebschner et al., 2019) and manual editing using COOT (Emsley et al., 2010) and ISOLDE (Croll, 2018).

The final models were validated using MolProbity ([Williams et al., 2018]; Table 1). The structural figures were rendered with PyMOL (The PyMOL Molecular Graphics System, Version 2.5 Schrödinger, LLC.) and USCF ChimeraX (Goddard et al., 2018; Pettersen et al., 2021).

**TABLE 1:** Cryo‐EM data collection, image processing, model building and validation statistics.

Sample	Wild‐type nucleosome (EMD‐39119, PDB ID: 8YBJ)	H3E97K nucleosome (EMD‐39120, PDB ID: 8YBK)
Data collection		
Electron microscope	Krios G4	Krios G4
Camera	K3	K3
Pixel size (Å/pix)	1.06	1.06
Defocus range (μm)	−1.0 to −2.5	−1.0 to −2.5
Exposure time (second)	4.5	4.5
Total dose (e^−^/Å^2^)	60	59
Movie frames (no.)	40	40
Total micrographs (no.)	7915	5806
Reconstruction		
Software	Relion 4.0	Relion 4.0
Particles for 2D classification	3,591,830	2,993,142
Particles for 3D classification	3,064,633	2,848,883
Particles in the final map (no.)	2,366,250	1,569,054
Symmetry	C1	C1
Final resolution (Å)	2.38	2.69
FSC threshold	0.143	0.143
Map sharpening B factor (Å^2^)	−69.2	−69.3
Model building		
Software	Coot	Coot
Refinement		
Software	Phenix, ISOLDE	Phenix, ISOLDE
Model composition		
Protein	758	708
Nucleotide	290	228
Validation		
MolProbity score	0.50	0.50
Clash score	0.00	0.00
R.m.s. deviations		
Bond lengths (Å)	0.0087	0.007
Bond angles (°)	0.961	0.994
Ramachandran plot		
Favored (%)	99.33	98.27
Allowed (%)	0.67	1.73
Outliers (%)	0.00	0.00

The atomic models and the cryo‐EM maps have been deposited in the Protein Data Bank and the Electron Microscopy Data Bank, under accession numbers 8YBJ and EMD‐39119 (the human wild‐type nucleosome), and 8YBK and EMD‐39120 (the H3E97K nucleosome).

### 3.8 Micrococcal nuclease digestion assay

The purified nucleosomes with the 145 base‐pair DNA (14.6 pmol) were incubated with micrococcal nuclease (0.7 units, MNase; Takara) at 37°C in 70 μL of reaction buffer (32 mM Tris–HCl (pH 7.5–8.0), 3.0 mM CaCl_2_, 26 mM NaCl, 2.1 mM dithiothreitol, and 2.5% glycerol) for 0, 3, 6, 9, 12, and 15 min. The reactions were terminated by mixing each 10 μL aliquot with 5 μL of deproteinization solution (20 mM Tris–HCl (pH 8.0), 0.1% sodium dodecyl sulfate [SDS], 20 mM ethylenediaminetetraacetic acid [EDTA], and 0.49 mg/mL proteinase K solution [Roche]). The samples were analyzed by 8% native‐PAGE, and the DNA fragments were visualized by EtBr staining. The gel images were acquired with an Amersham Imager 680 (Cytiva).

### 3.9 H1 binding assay

Human linker histone H1.2 was purified as a recombinant protein, as previously described (Machida et al., 2014). Increasing amounts (0–0.5 μM) of the linker histone H1.2 were mixed with the nucleosomes (0.1 μM) reconstituted with the 193 base‐pair DNA, in 10 μL of the reaction buffer (18 mM Tris–HCl (pH 7.5), 70 mM NaCl, 8% glycerol, 1.8 mM 2‐mercaptoethanol, 1.2 mM dithiothreitol, and 5 μg/μL bovine serum albumin [BSA]). The samples were incubated at 37°C for 30 min and analyzed by 5% native‐PAGE in 1× tris‐borate‐EDTA (TBE) buffer (90 mM Tris base, 90 mM boric acid, and 2 mM EDTA) with EtBr staining. The gel images were acquired using an Amersham Imager 680 (Cytiva). The band intensities corresponding to the unbound nucleosome were quantitated using the ImageQuant™ TL image analysis software (Cytiva).

### 3.10 PRC2 methyltransferase assay

The poly‐nucleosomes containing the wild‐type H3 or H3E97K mutant were prepared as described previously, with modifications (Dorigo et al., 2003; Kujirai et al., 2017). Briefly, the plasmid DNA containing 12 tandem repeats of the 208 base‐pair Widom 601 sequence (Lowary & Widom, 1998) was prepared and the DNA fragment containing the 12 repeats was isolated by *Eco*RV digestion, precipitated with polyethylene glycol, and subjected to TSKgel DEAE‐5PW column chromatography (TOSOH). The purified DNA fragment was mixed with the H2A‐H2B complex and H3‐H4 complex, and the poly‐nucleosomes were reconstituted by the salt dialysis method. The solution containing the poly‐nucleosomes was centrifuged for 10 min at 14,000*g* and 4°C to exclude aggregates. The resulting samples were further dialyzed against buffer, containing 10 mM Tris–HCl (pH 7.5), 1 mM dithiothreitol, and 5% glycerol, and stored at 4°C.

A *Sca*I cleavage site was inserted at every linker DNA region within the poly‐nucleosomes (Figure S7a). The nucleosome occupancy on the DNA fragment of the poly‐nucleosome was estimated by a *Sca*I cleavage assay. The poly‐nucleosome sample containing 100 ng DNA was incubated at 22°C for 12 h in the presence of ScaI (10 units) (Takara) in reaction buffer (10 mM Tris–HCl (pH 7.5), 0.5 mM MgCl_2_, 50 mM NaCl, and 0.1 mg/mL BSA). The resulting samples were fractionated by native‐PAGE and detected by EtBr staining. The gel image was captured using an Amersham Imager 680 (Cytiva).

To measure the PRC2 methyltransferase activity on the poly‐nucleosomes, we used the MTase‐Glo™ Methyltransferase Assay Kit (Promega). The poly‐nucleosome sample (34 nM) was mixed with the recombinant PRC2 complex (0.15 μM) (Active Motif #31387) in 10 μL of reaction solution (28 mM Tris–HCl (pH 7.5–8.5), 2.5 mM HEPES‐NaOH (pH 7.5), 40 mM NaCl, 0.2 mM 2‐mercaptoethanol, 0.6 mM dithiothreitol, 0.12 mM TCEP, 0.004% Triton X‐100, 3.5% glycerol, and 0.1 mM SAM). After an incubation at 30°C for 3 h, the reaction mixture was supplemented with 2 μL of 6× MTase‐Glo reagent and further incubated for 30 min at room temperature. The reaction solution was then mixed with 12 μL of MTase‐Glo Detection Solution and incubated for 30 min at room temperature. A 20 μL portion of the resulting reaction mixture was placed into a 384‐well plate (CORNING #4513). The luminescence of the samples was detected using a CLARIOstar Plus Microplate Reader (BMG LABTECH).

### 3.11 RNA polymerase II transcription assay

The *Komagataella pastoris* RNAPII and transcription elongation factor IIS (TFIIS) were prepared as previously described (Ehara, Umehara, et al., 2017; Ehara, Yokoyama, et al., 2017; Higo et al., 2014). The transcription reaction was conducted in the presence of the nucleosome containing the bubble DNA (0.1 μM), TFIIS (0.1 μM), RNAPII (0.1 μM), and DY647 fluorescently labeled RNA primer (0.4 μM) (5′‐DY647‐AUAAUUAGCUC‐3′) (Dharmacon) in 13 μL of the reaction solution (26 mM HEPES‐KOH (pH 7.5), 10 mM MgCl_2_, 0.2 μM zinc acetate, 50 mM potassium acetate, 0.1 mM dithiothreitol, 20 μM TCEP, 400 μM ATP, 400 μM GTP, 400 μM CTP, 400 μM UTP, and 1.5% glycerol) at 30°C. The reactions were terminated at 3, 6, 9, 12, and 15 min by mixing each aliquot (2 μL) with 2 μL of deproteinization solution (200 mM Tris–HCl (pH 8.0), 80 mM EDTA, and 0.5 mg/mL proteinase K [Roche]). After deproteinization, the samples were mixed with Hi‐Di formamide (Applied Biosystems), denatured for 10 min at 95°C, and fractionated by urea denaturing‐PAGE in 1 × TBE buffer (90 mM Tris base, 90 mM boric acid, and 2 mM EDTA). The DY647 fluorescence of RNA products was detected with an Amersham Typhoon imager (Cytiva). The band intensities corresponding to the run‐off products were quantified with the ImageQuant™ TL image analysis software (Cytiva).

## AUTHOR CONTRIBUTIONS

**Tomoaki Kimura:** Writing—original draft, Investigation. **Seiya Hirai:** Conceptualization, Writing—original draft, Investigation, Funding acquisition. **Tomoya Kujirai:** Conceptualization, Writing—review & editing, Funding acquisition. **Risa Fujita:** Investigation. **Mitsuo Ogasawara:** Investigation. **Haruhiko Ehara**: Investigation, Funding acquisition. **Shun‐ichi Sekine:** Investigation, Funding acquisition. **Yoshimasa Takizawa:** Writing—review & editing, Funding acquisition. **Hitoshi Kurumizaka:** Conceptualization, Writing—original draft, Writing—review & editing, Funding acquisition.

## FUNDING INFORMATION

This work was supported in part by JSPS KAKENHI grant numbers [JP20H03201 to H.E. and T.K., JP20H05690 to S.S. and T.K., JP22K15033 to T.K., JP22KJ0858 to S.H., JP22K06098 to Y.T., JP23K17392 to T.K., JP23H05475 to H.K., and JP24H02328 to H.K.]; Research Support Project for Life Science and Drug Discovery (BINDS) from AMED under grant number JP24ama121009 [to H.K.]; JST ERATO Grant Number JPMJER1901 [to H.K.].

## CONFLICT OF INTEREST STATEMENT

The authors declare that they have no known competing financial interests or personal relationships that could have appeared to influence the work reported in this paper.

## Supporting information


**Data S1.** Supporting Information.
