# Supplementary material for: Cryo‐EM structure and biochemical analyses of the nucleosome containing the cancer‐associated histone H3 mutation E97K
Source: Genes Cells. 2024 Jul 7;29(9):769–81. doi: 10.1111/gtc.13143 (PMC11448003; doi:10.1111/gtc.13143)
Supplement: Supplementary file 1 — Data S1. Supporting Information. [file GTC-29-769-s001.docx]

**Cryo-EM structure and biochemical analyses of the nucleosome containing the cancer-associated histone H3 mutation E97K**

Tomoaki Kimura^1,2^, Seiya Hirai^1,2^, Tomoya Kujirai^1^, Risa Fujita^1^, Mitsuo Ogasawara^1^, Haruhiko Ehara^3^, Shun-ichi Sekine^3^, Yoshimasa Takizawa^1^, and Hitoshi Kurumizaka^1,2,3*^

^1^Laboratory of Chromatin Structure and Function, Institute for Quantitative Biosciences, The University of Tokyo, 1-1-1 Yayoi, Bunkyo-ku, Tokyo 113-0032, Japan.

^2^Department of Biological Sciences, Graduate School of Science, The University of Tokyo, 1-1-1 Yayoi, Bunkyo-ku, Tokyo 113-0032, Japan.

^3^RIKEN Center for Biosystems Dynamics Research, 1-7-22 Suehiro-cho, Tsurumi-ku, Yokohama 230-0045, Japan.

^*^ **Corresponding author**: Correspondence should be addressed to H.Kurumizaka (e-mail: [kurumizaka@iqb.u-tokyo.ac.jp](mailto:kurumizaka@iqb.u-tokyo.ac.jp))

**
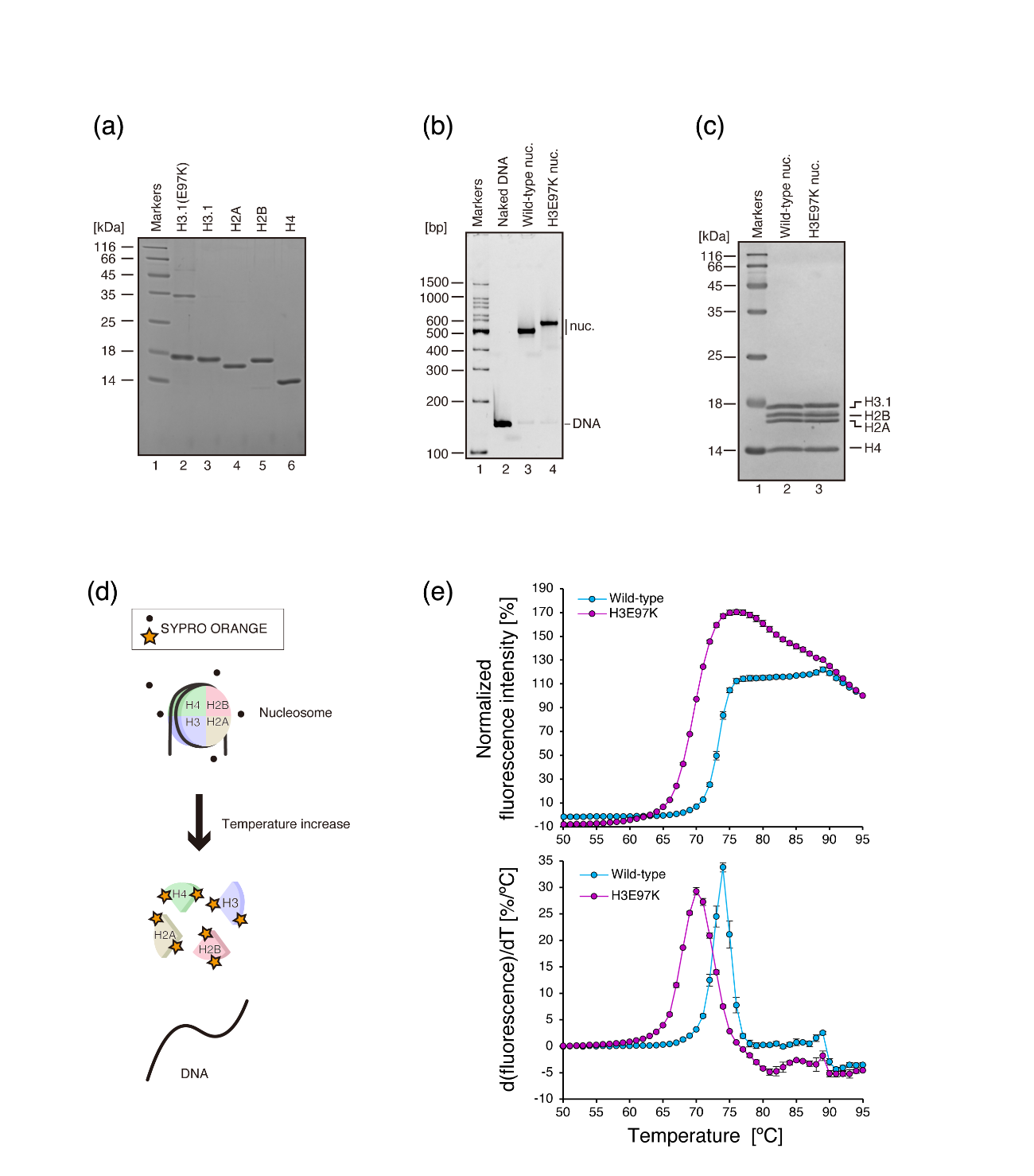
FIGURE S1. Structural instability of the H3E97K nucleosome.**

(a) The purified histones were analyzed by SDS-PAGE and stained with Coomassie Brilliant Blue (CBB). (b) The purified nucleosomes with the 145 base-pair DNA were analyzed by native-PAGE and stained with EtBr. (c) The histones in the purified nucleosomes with the 145 base-pair DNA were analyzed by SDS-PAGE and stained with CBB. (d) Schematic diagram of the thermal denaturation assay. The dissociation of the histone proteins from the nucleosomes by thermal denaturation was detected using SYPRO Orange fluorescent dye. (e) Thermal denaturation plots (upper panel) and derivative plots of thermal denaturation assay (lower panel). The error bars indicate standard deviations (n=3). Purple and blue circles indicate experiments with the H3E97K and wild-type nucleosomes, respectively.

**
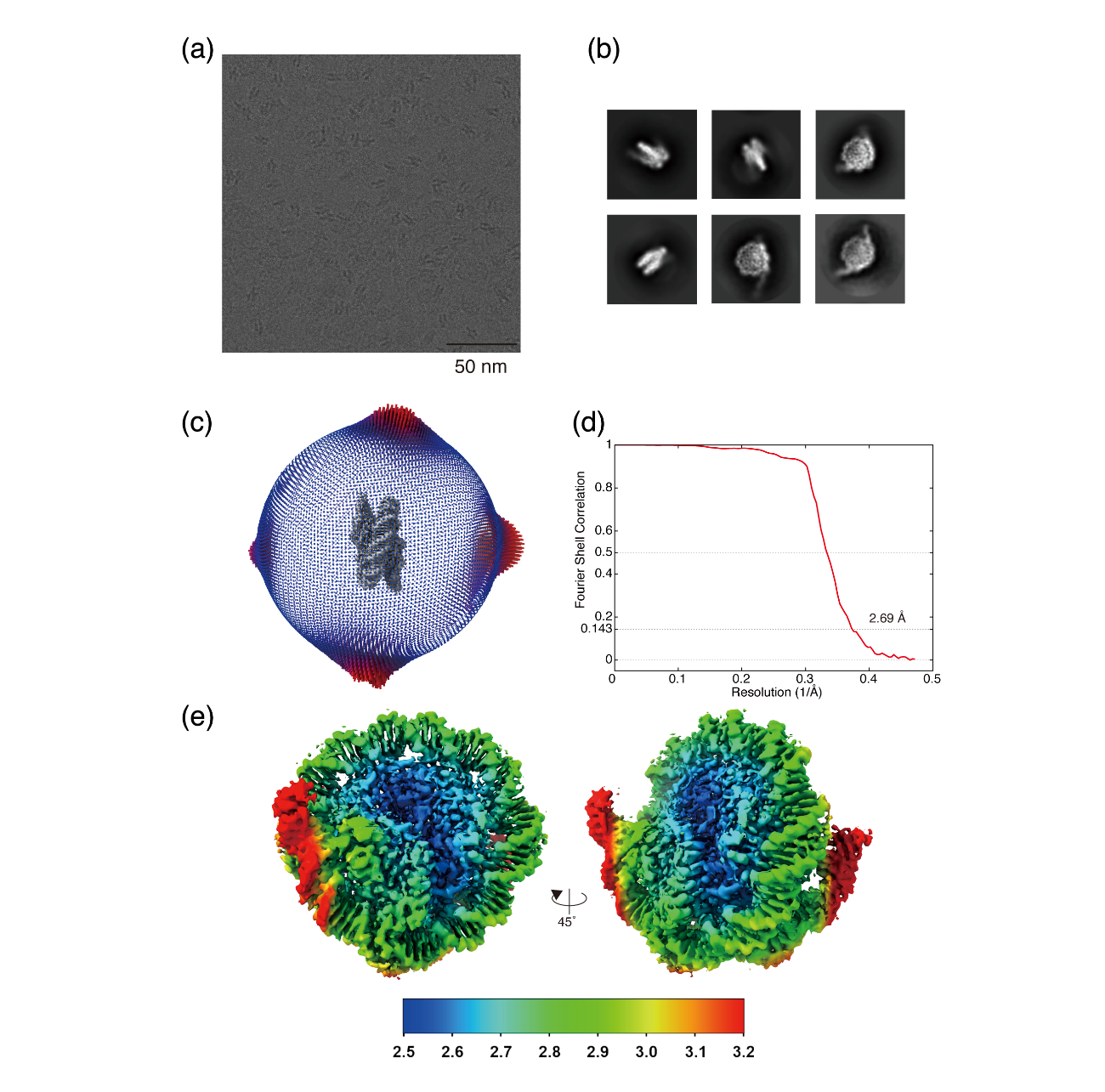
FIGURE S2. Cryo-EM analysis of the H3E97K nucleosome.**

(a) Representative micrograph of the H3E97K nucleosome. (b) Representative images of 2D class averages of the H3E97K nucleosome. (c) Euler angular distribution of the H3E97K nucleosome structure. (d) FSC curve of the H3E97K nucleosome structure. The resolution of the H3E97K nucleosome structure was estimated to be 2.69 Å (FSC = 0.143). (e) Local resolution map of the H3E97K nucleosome.

**
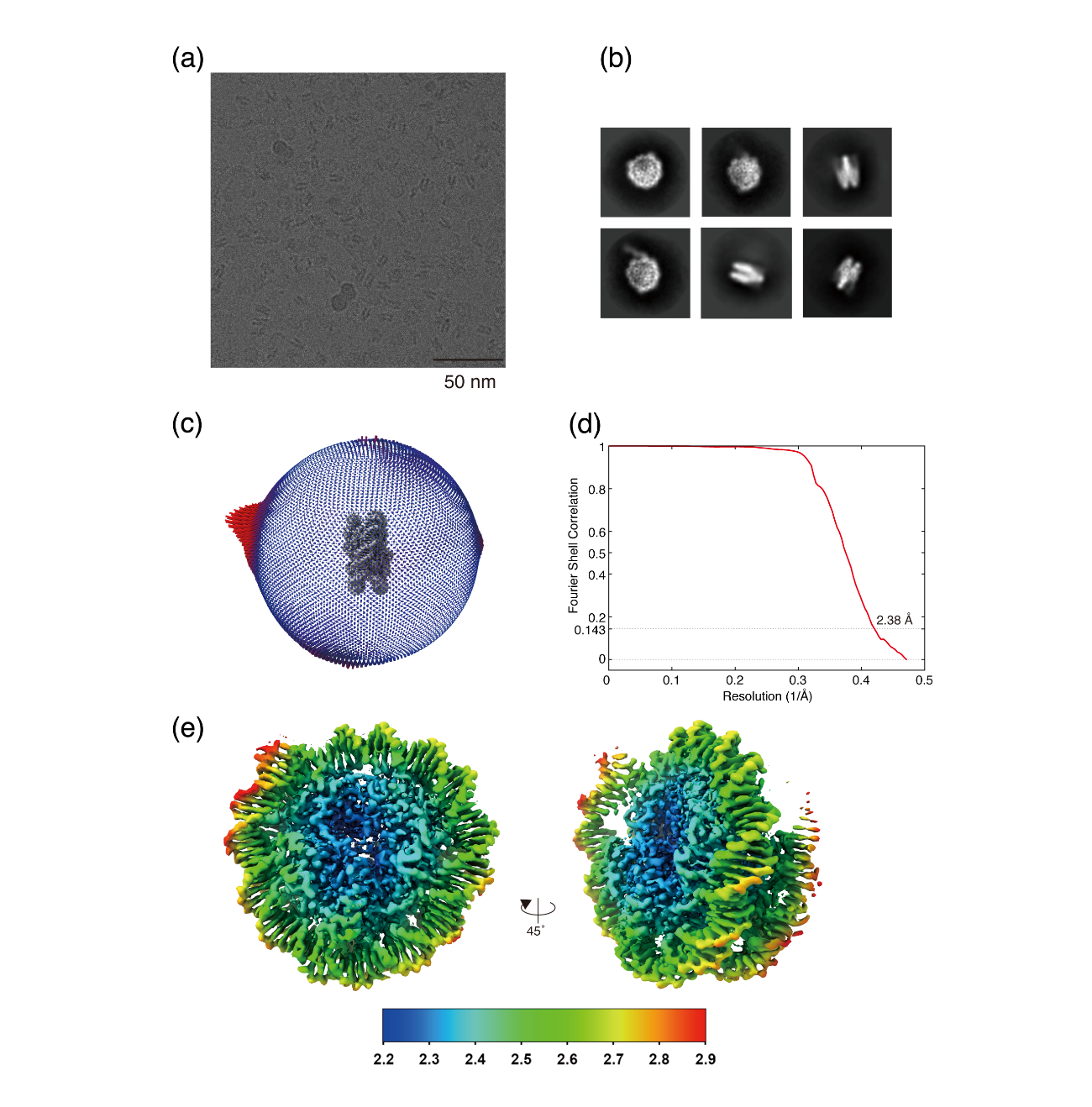
FIGURE S3. Cryo-EM analysis of the wild-type nucleosome.**

(a) Representative micrograph of the wild-type nucleosomes. (b) Representative images of 2D class averages of the wild-type nucleosomes. (c) Euler angular distribution of the wild-type nucleosome structure. (d) FSC curve of the wild-type nucleosome structure. The resolution of the wild-type nucleosome structure was estimated to be 2.38 Å (FSC = 0.143). (e) Local resolution map of the wild-type nucleosome.

**
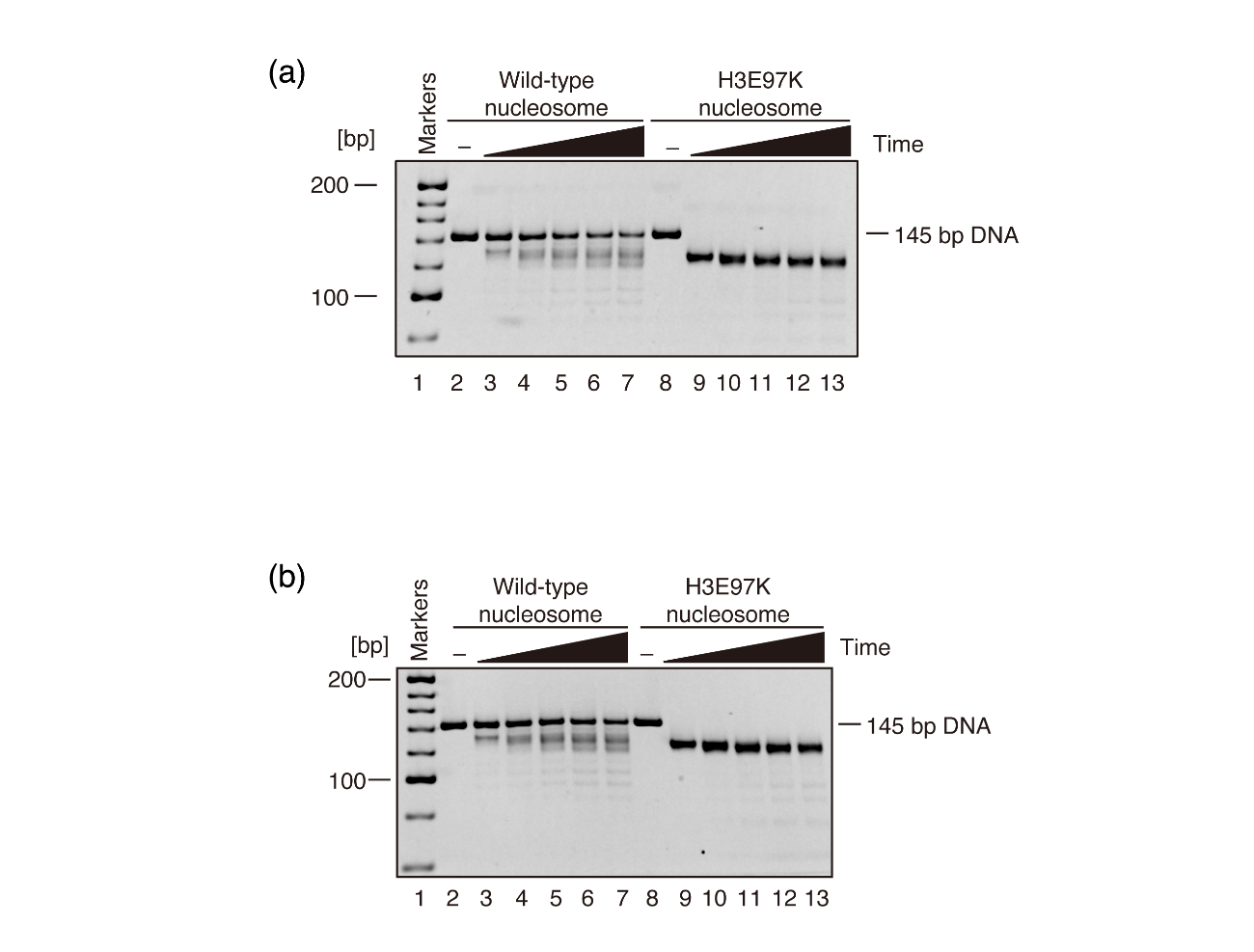
FIGURE S4.** (a, b) Replicates of MNase assay, as shown in Figure 1d.

**
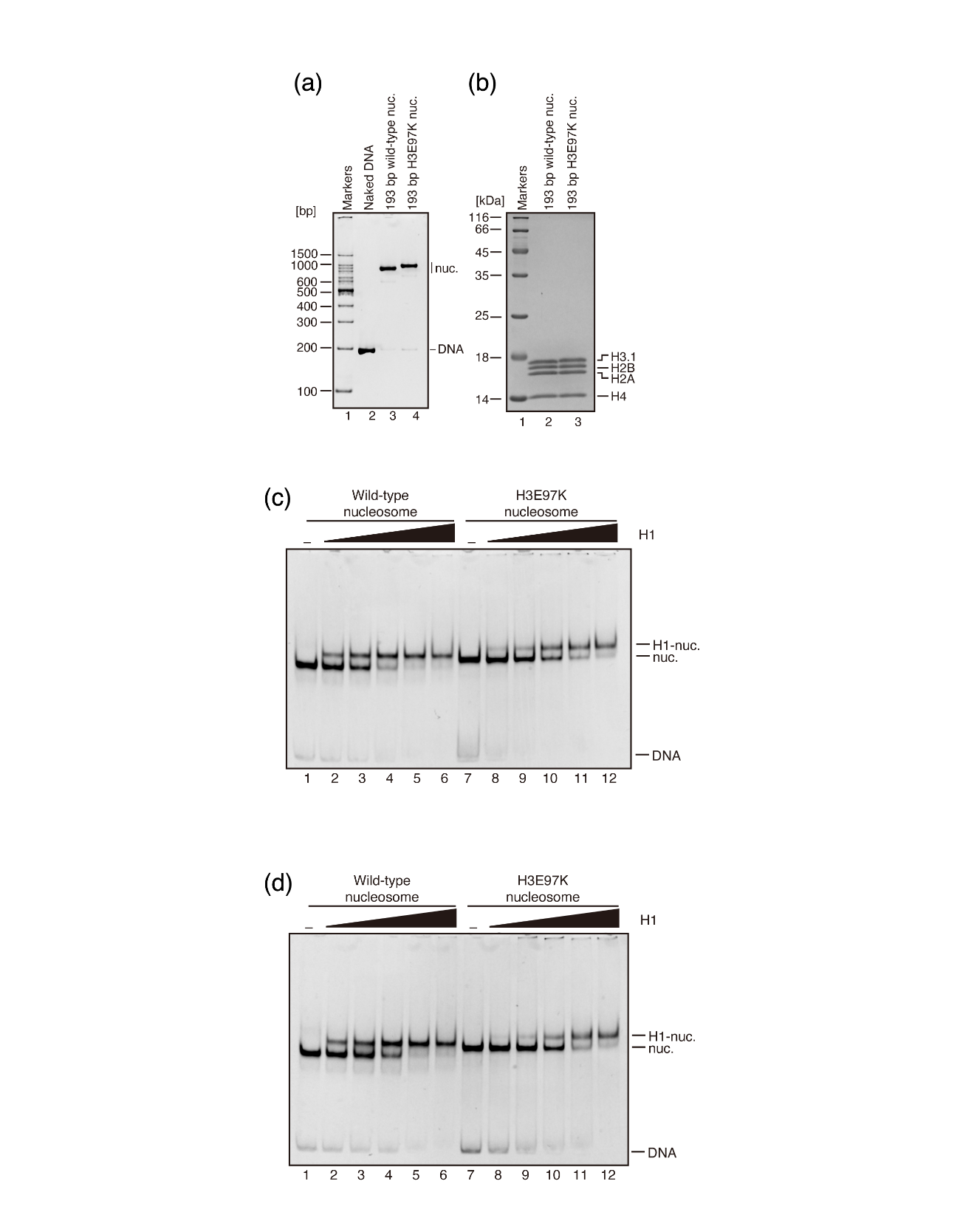
FIGURE S5.** (a) The purified nucleosomes with the 193 base-pair DNA were analyzed by native-PAGE and stained with EtBr. (b) The histones in the purified nucleosomes with the 193 base-pair DNA were analyzed by SDS-PAGE and stained with CBB. (c, d) Replicates of the H1 binding assay, as shown in Figure 3b.

**
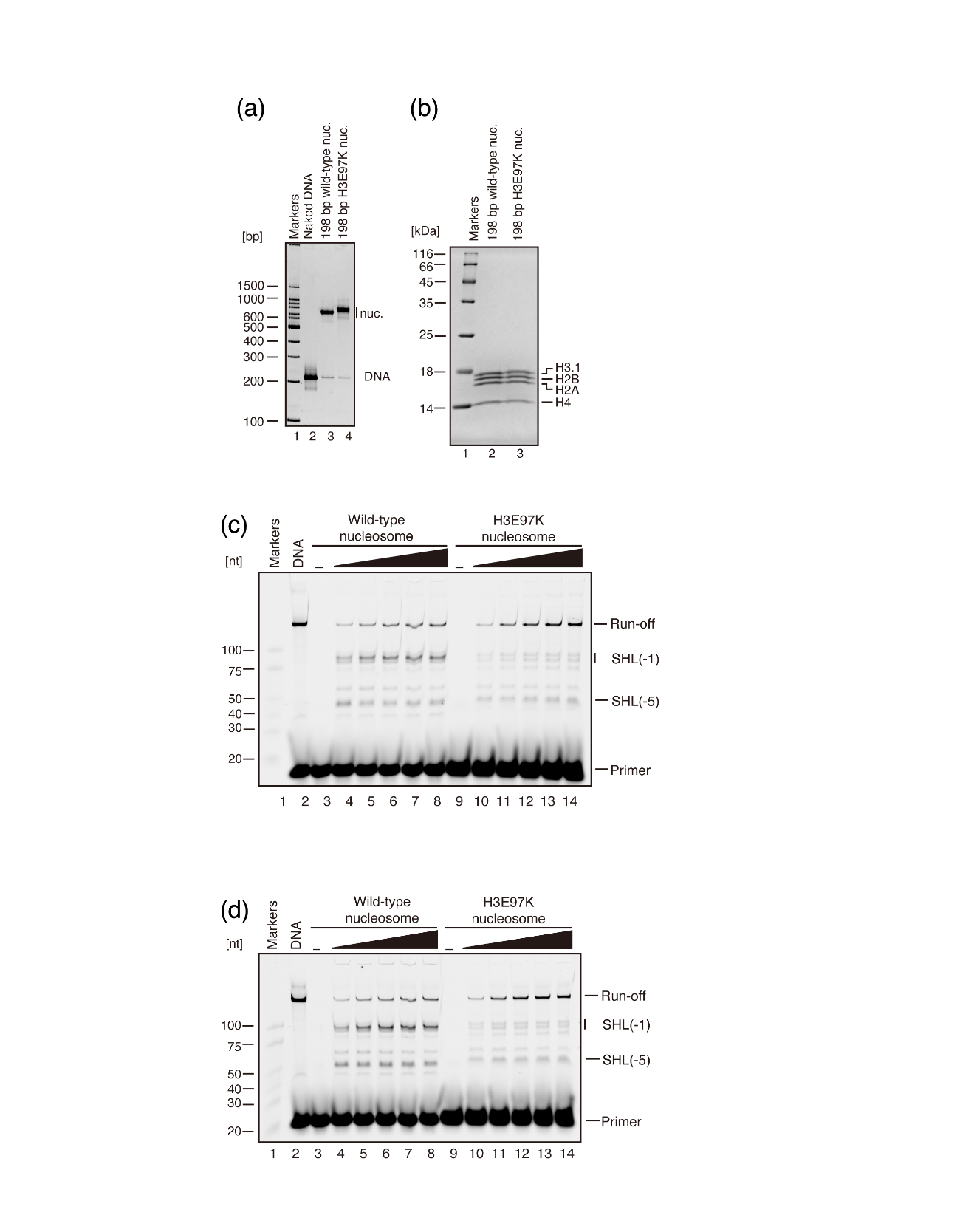
FIGURE S6.** (a) The purified nucleosomes with the 198 base-pair DNA were analyzed by native-PAGE and stained with EtBr. (b) The histones in the purified nucleosomes with the 198 base-pair DNA were analyzed by SDS-PAGE and stained with CBB. (c, d) Replicates of the RNA polymerase II transcription assay, as shown in Figure 5b.

**
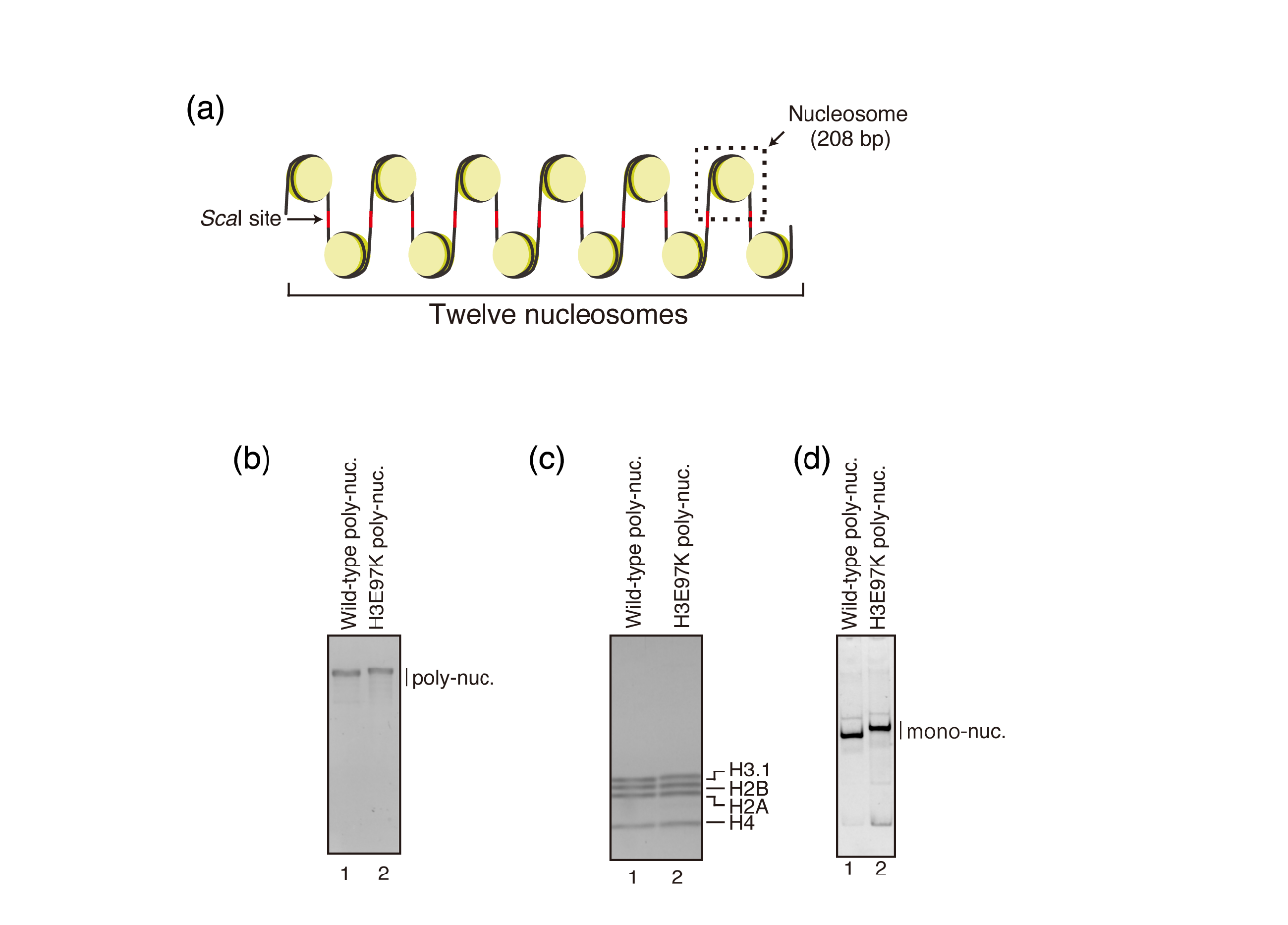
FIGURE S7.** (a) Schematic diagram of the poly-nucleosome used for the PRC2 methylation assay. The poly-nucleosomes were reconstituted with 12 repeats of the Widom 601 sequence (208 base-pairs). The red regions of the DNA indicate *Sca*I cleavage sites. (b) The purified poly-nucleosomes were analyzed by agarose gel electrophoresis and stained with EtBr. (c) The histones in the purified poly-nucleosomes were analyzed by SDS-PAGE and stained with CBB. (d) The *Sca*I cleavage assay of the poly-nucleosomes. The poly-nucleosomes were treated with the *Sca*I restriction enzyme and analyzed by native-PAGE with EtBr staining.
